# Surgery for *BRCA*, TP53 and PALB2: a literature review

**DOI:** 10.3332/ecancer.2018.863

**Published:** 2018-08-29

**Authors:** Chin-Vern Song, Soo-Hwang Teo, Nur Aishah Taib, Cheng-Har Yip

**Affiliations:** 1 Cancer Research Malaysia, Subang Jaya 47500, Malaysia; 2 University of Malaya, Kuala Lumpur 50603, Malaysia; 3 Ramsay Sime Darby Health Care, Subang Jaya 47500, Malaysia

**Keywords:** breast cancer, genetic mutations, surgery

## Abstract

**Introduction:**

The presence of a deleterious mutation, most commonly a *BRCA* mutation, has a tremendous impact on the management of breast cancer. We review the surgical management of *BRCA* mutation carriers, and two other potentially high-risk mutations, TP53 and PALB2.

**Methodology:**

A search was done on PubMed, limited to reviews and the English language only. The search terms used were ‘*BRCA*’ or ‘*PALB2*’ or ‘*TP53*’ and ‘surgery’. Fifteen articles were identified by searching and one article was obtained from other sources.

**Results:**

Breast-conserving surgery has equivalent survival, but may have an increased risk of local recurrence, compared to mastectomy among *BRCA* mutation carriers. Contralateral prophylactic mastectomy may not improve overall survival, despite reducing the risk of developing contralateral breast cancer. The use of preoperative genetic testing allows patients to have combined curative and prophylactic surgery. However, preoperative genetic testing may influence patients to make rash decisions. In healthy *BRCA* mutation carriers, bilateral prophylactic mastectomy is done to prevent breast cancer from occurring. Bilateral prophylactic mastectomy is highly effective in reducing the risk of breast cancer in healthy *BRCA* mutation-positive women and may have a survival benefit. Prophylactic oophorectomy reduces the risk of ovarian cancer, but may not have an effect on the risk of breast cancer. There is a lack of studies on surgery for non-*BRCA* mutations. *TP53* and *PALB2* are potentially high-risk mutations for breast cancer, which may justify the use of prophylactic surgery. Advice should be given on a case-by-case basis.

**Conclusion:**

A comprehensive approach is needed to provide optimum treatment for breast cancer patients with deleterious mutations.

## Introduction

Hereditary breast and ovarian cancer syndrome is associated with a higher risk of breast and ovarian cancer. This genetic syndrome is passed down in families, resulting in an increased incidence of cancer in affected families. The predisposing gene is only identified in 30% of cases, depending on family history and age of onset of disease [1].

The most commonly identified mutation is in the *BRCA1* and *BRCA2* genes. There are other less common mutations such as *TP53*, *PALB2*, *ATM* and *CHEK2* mutations which are associated with an increased risk of breast cancer. To date, much of the research on the clinical management of hereditary breast and ovarian cancer syndrome has focused on carriers of germline *BRCA1* or *BRCA2* mutations [1].

*BRCA1* was the first gene discovered to be associated with hereditary breast and ovarian cancer syndrome. *BRCA1* was discovered in 1994 and *BRCA2* was discovered in 1995. *BRCA1* is located on chromosome 17q while *BRCA2* is located on chromosome 13q [2]. *BRCA1* and *BRCA2* mutations are autosomal dominantly inherited. Both *BRCA* genes are involved in the repair of double-stranded DNA breaks. Since their discovery, hundreds of mutations have been discovered in the *BRCA* genes that predispose the individual to an increased risk of breast and ovarian cancer, such as *BRCA1*.185delAG, *BRCA1*.5382insC and *BRCA2.*6174delT in Ashkenazi Jews [1].

*BRCA* mutations are highly penetrant mutations. The absolute risk of breast cancer by the age of 80 years is 75% and 76% for protein truncating mutations of *BRCA1* and *BRCA2*, respectively, far higher than any other mutation associated with hereditary breast cancer. The magnitude of risk may differ according to the variant of the *BRCA* mutation present [3].

*BRCA1* mutation-associated breast cancer is more likely to be hormone receptor negative and have a higher grade and basal-like phenotype, while *BRCA2*-associated breast cancer resembles sporadic breast cancer [1]. However, *BRCA2*-associated breast cancer may be associated with poorer survival compared to sporadic breast cancer [4].

Apart from *BRCA* mutations, there are many other genetic mutations that may increase the risk of breast cancer. While the risk of breast cancer among *BRCA* mutation carriers is well-known and studied, the risk of breast cancer in other mutations are not well defined. Some mutations may confer a significant risk which may justify prophylactic surgery while others may carry only a small risk which may not warrant such extreme measures [3].

Surgical management previously did not take into account genetic mutation status. Now, with commercial multipanel genetic testing and a better understanding of the risk of disease, surgical management of hereditary breast cancer must now take into account the additional effects of deleterious mutations, such as increased risk of contralateral breast cancer and ovarian cancer in *BRCA* mutations. The types of surgery are the same as sporadic breast cancer, that is, a choice between a modified radical mastectomy and breast-conserving surgery in early stage breast cancer [5]. There is still controversy over the optimal surgical choice in *BRCA*-associated breast cancer with regards to the extent of breast tissue removed and the effectiveness of the different surgical techniques. Furthermore, there is the increasing use of prophylactic bilateral mastectomy among healthy women with *BRCA* mutations to prevent future breast cancer, while *BRCA* mutation carriers with breast cancer are also choosing to have a contralateral prophylactic mastectomy to prevent contralateral breast cancer [6].

This is a review of the literature on the current surgical treatment for *BRCA* mutation carriers. We also review *TP53* and *PALB2* mutations, as both of these genes are associated with a potentially high risk of breast cancer [3].

## Methodology

A literature review was done on PubMed on surgery in hereditary breast and ovarian cancer syndrome. Keywords used were: ‘*BRCA*’ or ‘*PALB2*’ or ‘*TP53*’ and ‘surgery’. The maximum date for articles was April 2018. Articles were limited to reviews and English language only. Journal articles were selected subjectively based on whether the subject was relevant to the use of surgery in *BRCA* or non-*BRCA* hereditary breast or ovarian cancer syndrome. The first author was involved in selecting and determining the suitability of articles.

Three-hundred and sixty-one articles were found by searching on PubMed. Articles were then chosen based on whether the title was relevant to this review. After eliminating articles with irrelevant titles, 68 articles were remaining. The 68 articles were selected for examination of the abstract. After eliminating irrelevant articles, 15 articles were remaining. The text was then examined for relevance. No articles had irrelevant text. Fifteen articles were selected to be included in this review.

Most of the articles were on *BRCA* mutations. There was a lack of studies on other mutations. Only one study was focused on breast cancer in women with *TP53* mutations. No studies could be found exclusively focusing on breast cancer in women with *PALB2* mutations.

Overall, 19 articles were included in this review (Table 1). Fifteen were obtained from searching PubMed as mentioned earlier. Four studies (Easton *et al* [3], Li *et al* [13], Lostumbo *et al* [16] and D’souza *et al* [19]) were obtained from other sources.

### Limitations

Articles were selected based on the first author’s opinion of whether they were relevant or not. No objective criteria were used to determine relevance. The subjective interpretation of a single author is an acknowledged flaw of this study.

## Results

### Breast-conserving surgery and mastectomy have equivalent outcomes in BRCA mutation carriers and non-carriers

Given that carriers of germline *BRCA1* or *BRCA2* alterations are more likely to develop breast cancer, it is arguable that mastectomy may be indicated instead of breast-conserving surgery in all carriers to prevent local recurrence in the remaining breast tissue. However, a review by Biglia *et al* [7] found no significant difference in overall survival between breast-conserving surgery and mastectomy among *BRCA* mutation carriers at 15 years. However, at 15 years after surgery, the risk of local recurrence was 23.5% versus 5.5% for breast-conserving surgery and mastectomy, respectively [7].

If no difference is seen in overall survival between mastectomy and breast-conserving surgery among *BRCA* mutation carriers, then what is the outcome of breast-conserving surgery in *BRCA* mutation carriers compared to non-carriers? The review by Biglia *et al* [7] also found that there was no difference in overall survival between *BRCA* mutation carriers and non-carriers who had breast-conserving surgery, but *BRCA* mutation carriers may have an increased risk of local recurrence in the long term. A meta-analysis by Valachis *et al* [8] found no significant difference in local recurrence between *BRCA* mutation carriers and non-carriers. However, when analysing studies with a median follow up of 7 or more years, there was a significant increase in local recurrence (Relative risk: 1.51).

The phenomenon of late local recurrence in both cases could be due to new primary tumours forming in the remaining breast tissue rather than a true local recurrence of the malignancy [9]. As new primary cancers tend to be less aggressive and without treatment resistance compared to true local recurrences, this may explain why *BRCA* carriers who had breast-conserving surgery may experience a higher risk of local recurrence but no difference in overall survival [9].

Radiotherapy is an important component in breast-conserving surgery to reduce the risk of local recurrence. Previously, there was concern over the safety of radiotherapy in *BRCA* mutation carriers, as *BRCA* mutation carriers have impaired DNA repair mechanisms. However, a review by Pierce and Haffty [10] found no impaired survival and no increase in adverse effects due to the use of radiotherapy in *BRCA* mutation carriers. The lack of evidence of harm for radiotherapy indicates that it may be safe in *BRCA* mutation carriers and should not be withheld.

### Contralateral prophylactic mastectomy in BRCA positive breast cancer patients

*BRCA*-positive breast cancer patients have an increased risk of contralateral breast cancer compared to non-carriers. A review by Biglia *et al* [7] found that the risk of metachronous contralateral breast cancer was as high as 27% for *BRCA*1 mutation and 19% for *BRCA*2 mutation at 10 years after initial surgery. In contrast, the risk of contralateral breast cancer at 10 years after treatment in non-carriers was only 5%. Regular screening is recommended to detect new cancers early when the prognosis is better, but cannot prevent new cancers from forming. The effect of chemoprevention of contralateral breast cancer with selective oestrogen receptor modulators or adjuvant chemotherapy in *BRCA* mutation carriers is not clear, with inconsistent results between studies and oophorectomy as a confounding factor providing little clarity [9]. As such, contralateral prophylactic mastectomy can be considered to reduce the risk of contralateral breast cancer in *BRCA* mutation carriers who have already developed breast cancer on one side. The procedure can be performed with the curative surgery, or after primary surgery.

Contralateral prophylactic mastectomy shows an increasing trend in the Western world. Use of contralateral prophylactic mastectomy is more common among Caucasians, younger women and those of high socioeconomic status. The usage of more advanced diagnostic methods, such as magnetic resonance imaging or genetic testing, is associated with contralateral prophylactic mastectomy [11]. This could be due to an increased level of testing causing more fear and anxiety to patients, which may push them towards more radical surgery. The increasing availability of breast-reconstructive surgery is a predictor of having contralateral prophylactic mastectomy [11]. The availability of breast reconstructive surgery could also improve the cosmetic outcome of the procedure, which improves patient satisfaction, hence patients would be more willing to undergo contralateral prophylactic mastectomy [7].

Despite the growing popularity of contralateral prophylactic mastectomy, its effects on survival are less clear. A meta-analysis by Fayanju *et al* [12] found that contralateral prophylactic mastectomy decreased the risk of metachronous contralateral breast cancer, but did not have an effect on overall survival among patients with elevated familial or genetic risk. However, a meta-analysis led by Li *et al* [13] shows a different result. Contralateral prophylactic mastectomy was associated with a decrease in all-cause mortality in the meta-analysis.

Currently, there are no prospective randomised controlled trials comparing contralateral prophylactic mastectomy with unilateral surgery, nor is it likely there will ever be one [11].

Although mastectomy is known to have more psychosocial adverse effects than breast-conserving surgery, women who opt for contralateral prophylactic mastectomy are reported to be highly satisfied with their decision, with only 5% experiencing regret. The majority do not experience body image issues, sexual issues or emotional distress [7]. For women that experience regret, the main reason was the poor cosmetic outcome, followed by reduced sexuality and lack of knowledge of alternatives to contralateral prophylactic mastectomy [9].

### Treatment-focused genetic testing and combined curative and prophylactic surgery in BRCA-positive breast cancer patients

Previously, *BRCA* mutation testing was typically conducted after completion of treatment for breast cancer, and therefore, prophylactic contralateral mastectomy was typically not offered at the time of treatment for primary breast cancer. With the development of rapid *BRCA* testing, patients have the opportunity to know their *BRCA* mutation status before primary surgery, and such treatment-focused genetic testing provides *BRCA* carriers with the opportunity to reduce the need for separate surgeries for treatment and prophylactic prevention [9].

Indeed, with the advent of such genetic testing, *BRCA* carriers are less likely to choose breast conservation compared to non-carriers. Women who test positive for a *BRCA* mutation are more likely to have more radical surgery and even bilateral mastectomy [9]. However, in the event of a prognosis such as advanced disease or existing metastasis, bilateral mastectomy is not recommended, as it offers little benefit [14].

With the advent of preoperative genetic testing, new issues arise with combining treatment-focused surgery and prophylactic surgery. For example, there may be insufficient time to understand the information about risk, leading to a rash, extreme decision made in the heat of the moment. Even with a negative test result, more than half of patients still chose to have a contralateral prophylactic mastectomy in one study [11]. In addition, without sufficient time to make a considered decision, there may be a higher risk of longer-term feelings of regret. To date, no studies have compared satisfaction in combined curative and prophylactic surgery, compared to a two-step surgical management strategy.

### Bilateral prophylactic mastectomy in BRCA carriers without breast cancer

Genetic testing can also be done for healthy individuals, not just those who have already developed cancer. Once a healthy woman knows she is positive for a *BRCA* mutation, she can either have regular screening or go for risk-reducing surgical methods, such as bilateral prophylactic mastectomy. While contralateral prophylactic mastectomy is done in *BRCA* carriers who have already developed breast cancer as a means to reduce the risk of contralateral breast cancer, bilateral prophylactic mastectomy involves removal of both healthy breasts to prevent breast cancer from developing in the first place as a form of primary prevention.

According to a review led by Wainberg and Husted [15] in 2004, the rates of prophylactic mastectomy range from 0% to 54% in unaffected women in the USA and The Netherlands. The rate of bilateral prophylactic mastectomy was noted to be higher in the Netherlands compared to the USA (47%–54% versus 0%–15%) [15]. The decision to have a bilateral prophylactic mastectomy is an individual decision which may be affected by culture and personal beliefs, which may explain the wide range in rates of prophylactic mastectomy.

Bilateral prophylactic mastectomy is able to reduce the risk of breast cancer by more than 95%. This translates into reduced breast cancer-specific mortality [14]. A systematic review by Lostumbo *et al* [16] found that bilateral mastectomy was able to reduce deaths from breast cancer. However, a meta-analysis by Li *et al* [13] found that bilateral prophylactic mastectomy does not significantly affect all-cause mortality.

However, there are few issues with bilateral prophylactic mastectomy. One issue is the optimal timing for bilateral prophylactic mastectomy. There is no fixed time limit for bilateral prophylactic mastectomy; it can be done as soon as genetic testing is complete, or delayed indefinitely. However, one must take into consideration that breast cancer in *BRCA1* mutation carriers develops at a significantly younger age, about 10 years younger than sporadic breast cancer [17]. Therefore, it is prudent to discuss the option of prophylactic surgery at the moment of discovering *BRCA* mutation status.

The effect of bilateral prophylactic mastectomy on quality of life is another issue. A review led by Razdan *et al* [18] found that the majority of patients (70%) were satisfied with the surgery. Body image was not found to be negatively affected, with the majority (65%) of women maintaining a positive body image following prophylactic mastectomy. More than 60% of women reported favourable sexual well-being following surgery, although many reported loss of sensation in the breast. 95% of women did not experience regret following bilateral prophylactic mastectomy, indicating high levels of confidence with their decision [18]. Overall, patient satisfaction with bilateral prophylactic mastectomy is high, similar to that observed with contralateral prophylactic mastectomy. The high satisfaction could be due to patients experiencing less anxiety over breast cancer after the procedure [14].

Another issue is the type of surgery used in a bilateral prophylactic mastectomy. The goal is to remove enough breast tissue for optimal risk reduction, as well as provide an aesthetically acceptable outcome with the help of reconstructive surgery. Skin-sparing and nipple-sparing mastectomies are also considered safe. The risk of malignancy following conservation of the areola is 3.5%–5.5% in 5–7 years [14]. It is likely that different types of mastectomy may have different effects on patient satisfaction. More studies are needed to discern this issue.

The cost-effectiveness of bilateral prophylactic mastectomy in *BRCA* mutation carriers is another issue. Despite the increased risk of breast cancer, not all *BRCA* mutation carriers will develop breast cancer. Whether the use of bilateral prophylactic mastectomy among *BRCA* mutation carriers is more cost-effective than surveillance and early detection of breast cancer is not known. This issue is not well studied in the literature. More studies on this issue are needed to get a clearer view on the cost-effectiveness of prophylactic mastectomy.

### Psychological burden of more extensive surgery

The combination of prophylactic surgery, therapeutic surgery or breast reconstruction in one operation leads to a more extensive and longer single surgery, although it reduces the need for two operations. The psychological burden of this is not well understood. A systematic review on the effect of immediate or delayed breast reconstruction was inconclusive on the impact of more extensive surgery on psychosocial issues [19].

### Risk-reducing salpingo-oophorectomy

Hereditary breast and ovarian cancer syndrome also carries the increased risk of ovarian cancer. The cumulative risk for developing ovarian cancer is estimated to be 39% and 11% for *BRCA*1 and *BRCA*2 mutation carriers, respectively. *BRCA*1 mutation carriers tend to develop ovarian cancer at a younger age compared to *BRCA*2 mutation carriers [20].

Unlike breast cancer, there is no effective screening method for ovarian cancer. The current screening methods of serum CA125 measurement and regular transvaginal ultrasonography cannot reliably detect ovarian cancer early [21]. Therefore, ovarian cancer in *BRCA* mutation carriers tends to be discovered at an advanced stage with poor prognosis. Due to the lack of effective screening methods, prophylactic surgery must be considered. Bilateral salpingo-oophorectomy is a risk-reducing surgical method to reduce the risk of ovarian cancer in *BRCA* mutation carriers.

According to National Comprehensive Cancer Network guidelines, the procedure is recommended in *BRCA* mutation carriers around the age of 35–40 years old or when childbearing is complete. *BRCA*2 mutation carriers may be able to delay surgery up to the age of 45 years, as *BRCA*2 mutation carriers tend to develop ovarian cancer after the age of 50 years. The guidelines do not recommend screening, as there is a lack of evidence for the effectiveness of screening [20].

Bilateral salpingo-oophorectomy can reduce the risk of ovarian cancer in healthy *BRCA* mutation carriers by over 80%. The few remaining cases of ovarian cancer can arise from primary peritoneal cancer. Apart from reducing the risk of developing ovarian cancer, prophylactic oophorectomy can significantly decrease the all-cause mortality as well [21]. There may be a difference in protective effect according to the type of *BRCA* mutation. A meta-analysis showed that *BRCA*2 mutation carriers did not have a significant reduction in risk of ovarian cancer while women with *BRCA*1 mutation did. However, all-cause mortality was similarly reduced for both *BRCA*1 and *BRCA*2 mutation carriers [22].

The effect of prophylactic oophorectomy on breast cancer is less clear. It was previously thought that prophylactic oophorectomy could reduce the risk of breast cancer in *BRCA* mutation carriers by up to 50%, particularly in younger patients. Recently, two large prospective studies found no statistically significant reduction in risk of breast cancer after prophylactic oophorectomy [21]. Previous studies on this issue have been mostly retrospective, which could suffer from bias.

The main drawback of prophylactic oophorectomy is surgically induced menopause. Women who undergo prophylactic oophorectomy have increased risk of coronary heart disease, osteoporosis, obstructive lung disease, diabetes, arthritis and mental health issues. Sexual functioning is also adversely affected [21]. The use of hormone replacement therapy to control symptoms of surgically induced menopause is controversial. It has been suggested that hormone replacement therapy may further increase the risk of breast cancer in BRCA mutation carriers, along with increasing the risk of endometrial cancer. There is limited data available on the safety of hormone replacement therapy in BRCA mutation carriers after prophylactic oophorectomy [21]. Hormone replacement therapy should be used with caution after prophylactic oophorectomy, as there is no definite evidence of its safety.

### Surgery in non-BRCA hereditary breast and ovarian cancer syndrome

While *BRCA* mutations are the most common and well-studied in hereditary breast and ovarian cancer syndrome, there are other much rarer genetic mutations associated with familial breast cancer. For the majority of these presumed breast cancer genes, risk estimates remain uncertain [3]. The use of prophylactic surgery is more variable, as there is very little evidence of the effectiveness of prophylactic surgery for many of the mutations. It is recommended that physicians refer to pre-existing guidelines and consider the patient’s family history before giving advice, until more evidence and better estimates of absolute risk are available. Here, we will focus on two specific gene mutations, *TP53* and *PALB2*, as both these genes are associated with a potentially high risk of breast cancer [3].

Germline *TP53* mutations are associated with Li Fraumeni syndrome. The absolute risk of breast cancer in *TP53* mutation carriers is not known, as most studies on *TP53* mutation carriers suffer from ascertainment bias [3]. Patients with this syndrome are more likely to develop breast cancers at a younger age. The median age of diagnosis of breast cancer in *TP53* mutation carriers is 34 years old. Breast cancers associated with the *TP53* mutation tend to be hormone-receptor positive and HER2 positive. Bilateral mastectomy can be reasonably recommended for both healthy carriers and those with breast cancer, due to the high risk of developing breast cancer. Use of breast-conserving surgery is not recommended, as *TP53* mutation carriers are more susceptible to radiation-induced DNA damage and are more likely to develop radiation-associated cancers such as angiosarcoma [23].

*PALB2* is another gene mutation associated with hereditary breast cancer. Women with *PALB2* mutations have up to a fivefold increase in the risk of developing breast cancer compared to the general population. However, the confidence interval is too wide to be certain of the risk [3]. Currently, there is a lack of studies on the surgical treatment of women with this mutation. No studies that focused exclusively on surgery for *PALB2* mutation carriers could be found on searching. The possibly high risk of breast cancer makes bilateral prophylactic mastectomy a potential option for women with *PALB2* mutations. A case-by-case approach based on family history before recommending prophylactic mastectomy is reasonable in this group of patients [14].

For the use of mastectomy or breast-conserving surgery in other non-*BRCA* hereditary breast and ovarian cancer syndrome, there is very little literature available. The use of radiotherapy is a major concern, as there is a lack of evidence for or against the use of radiotherapy in many non-*BRCA* mutations. Although *BRCA* mutations are safe in radiotherapy as mentioned earlier, the effects of radiotherapy on other mutations are not well understood [10]. Some mutations such as *TP53* may even have an increased risk of adverse events [23]. Therefore, it would be prudent to avoid breast-conserving surgery until the safety of radiotherapy in the specific mutation is established.

## Conclusion

*BRCA* mutations present a challenge in management. Apart from the high risk of initial breast cancer, patients are faced with the risk of new primary breast cancer/local recurrence, ovarian cancer as well as the psychological burden of the high risk of cancer. Surgical management must be modified to provide optimal treatment for *BRCA* mutation carriers. Breast-conserving surgery is generally safe in *BRCA* mutation carriers, as it does not negatively affect survival, but the risk of local recurrence may be increased in the long term. Contralateral prophylactic mastectomy may not be able to improve overall survival, despite reducing the risk of contralateral breast cancer. Bilateral prophylactic mastectomy for healthy *BRCA* mutation carriers is an effective risk-reducing method that may be able to improve long-term survival and alleviate the fear of breast cancer. The option of breast reconstruction must be offered in all women undergoing a risk-reducing mastectomy. Other options such as intensive surveillance should be offered to those who defer surgery, with a clear explanation that these modalities can only downstage the disease, and not prevent it.

Most of the literature for surgery in hereditary breast cancer has been on those with *BRCA* mutations. The effect of other mutations associated with hereditary breast and ovarian cancer on surgical decisions is not well studied. Due to the lack of data available, there are no definite surgical recommendations in non-*BRCA* hereditary breast and ovarian cancer syndrome.

## Conflicts of interest

There are no conflicts of interest to declare.

## Funding

This review received no funding.

## Figures and Tables

**Table 1.** Selected studies for systematic review.

First author	Year	Methodology	Title	Finding
Valachis	2014	Systematic review and meta-analysis	Surgical management of breast cancer in *BRCA*-mutation carriers: a systematic review and meta-analysis	Breast-conserving surgery is safe in *BRCA* mutation carriers but increases risk of local recurrence ≥7 years Increased risk of contralateral breast cancer among *BRCA* mutation carriers
Nestle-Krämling	2012	Literature review	Role of breast surgery in *BRCA* mutation carriers	Bilateral prophylactic mastectomy reduces the risk of breast cancer by 90%–95% High satisfaction with prophylactic surgery. 90% willing to still do it even 20 years post surgery Prophylactic surgery rates vary worldwide even among Western countries (32.7% in the Netherlands versus 2.7% in Poland)
Smith	2011	Literature review	*BRCA* mutation testing in determining breast cancer therapy	Preoperative genetic testing affects surgery choice and is not associated with increased distress Breast-conserving surgery may have increased risk long term of local recurrence Most women do not experience regret after prophylactic mastectomy. Reported reasons for regret include poor cosmetic result, reduced sense of sexuality and lack of education about contralateral prophylactic surgery versus screening Oophorectomy is recommended for women with *BRCA-*associated breast cancer aged 35–40 who have completed childbearing Oophorectomy reduces the risk of ovarian cancer in *BRCA* mutation carriers by at least 80%–90% and may reduce the risk of breast cancer
Fayanju	2014	Systematic review and meta-analysis	Contralateral prophylactic mastectomy after unilateral breast cancer: a systematic review and meta-analysis	Contralateral prophylactic mastectomy reduces the risk of contralateral breast cancer among patients with genetic risk but does not affect survival
Biglia	2016	Literature review	Breast cancer treatment in mutation carriers: surgical treatment	Breast-conserving surgery may have an increase risk of local recurrence >15 years but no effect on overall survival *BRCA* mutation has a higher risk of contralateral breast cancer No difference in overall survival if Contralateral prophylactic mastectomy was done.
Levine	2003	Literature review	Prophylactic surgery in hereditary breast/ovarian cancer syndrome	Contralateral prophylactic mastectomy does not increase overall survival No evidence for performing sentinel lymph node biopsy in prophylactic mastectomy in healthy women 5%–30% of bilateral prophylactic mastectomy patients experience regret, perceived physician centred decision making increases the risk. Patients may overestimate their risk of breast cancer. 86% of women highly satisfied with prophylactic oophorectomy. Less disfigurement issues with prophylactic oophorectomy compared to mastectomy
Wainberg	2004	Systematic review	Utilisation of screening and preventive surgery among unaffected carriers of a *BRCA*1 or *BRCA*2 gene mutation	Rates of prophylactic mastectomy range from 0% to 54% and prophylactic oophorectomy range from 13% to 53%. Variable rates of prophylactic surgery even within same populations
Razdan	2016	Systematic review	Quality of life among patients after bilateral prophylactic mastectomy: a systematic review of patient-reported outcomes	Bilateral prophylactic mastectomy patients are satisfied with their decision (61%–100%)
Yao	2016	Literature review	Contralateral prophylactic mastectomy: current perspectives	Contralateral prophylactic mastectomy rates increasing in the US No effect of Contralateral prophylactic mastectomy on survival Women satisfied with Contralateral prophylactic mastectomy
Neff	2017	Literature review	*BRCA* mutation in ovarian cancer: testing, implications and treatment considerations	Prophylactic oophorectomy is associated with a decreased risk of ovarian cancer. There is still a small risk of primary peritoneal cancer Ovarian cancer screening is not effective
Mau	2017	Literature review	Prophylactic surgery: for whom, when and how	Bilateral prophylactic mastectomy can reduce the incidence of breast cancer and improve survival
Pierce	2011	Literature review	Radiotherapy in the treatment of hereditary breast cancer	No evidence of decreased survival or increased adverse events of radiotherapy in *BRCA* mutation carriers Lack of evidence for radiotherapy in other rare mutations
Marchetti	2014	Systematic review and meta-analysis	Risk-reducing salpingo-oophorectomy: a meta-analysis on the impact on ovarian cancer risk and all-cause mortality in *BRCA* 1 and *BRCA* 2 mutation carriers	Meta-analysis showed a reduction in the risk of ovarian cancer among *BRCA* mutation carriers
Tschernichovsky	2017	Systematic review	Risk-reducing strategies for ovarian cancer in *BRCA* mutation carriers: A balancing act	Prophylactic oophorectomy is effective at reducing the risk of ovarian cancer but has significant adverse effects
Schon	2018	Literature review	Clinical implications of germline mutations in breast cancer: *TP53*	*TP53* is associated with a high risk of developing hormone-receptor positive breast cancer in young women
Easton	2015	Meta-analysis	Gene-panel sequencing and the prediction of breast cancer risk	Apart from *BRCA*1/2, reliable estimates of risk are lacking for most other genes associated with hereditary breast cancer
D’Souza	2011	Systematic review	Immediate versus delayed reconstruction following surgery for breast cancer	Psychological impact of more extensive surgery is not well known
Li	2016	Systematic review and meta-analysis	Effectiveness of prophylactic surgeries in BRCA1 or BRCA2 mutation carriers: a meta-analysis and systematic review	Contralateral prophylactic mastectomy significantly reduces mortality while bilateral prophylactic mastectomy does not
Lostumbo	2010	Systematic review	Prophylactic mastectomy for the prevention of breast cancer	Bilateral mastectomy is able to reduce deaths from breast cancer Effect of contralateral prophylactic mastectomy on survival is inconclusive
